# The Impact of Type II Diabetes on Tongue Dysplasia and p16-Related Aging Process: An Experimental Study

**DOI:** 10.1155/2019/3563215

**Published:** 2019-10-08

**Authors:** Eren Altun, Hasmet Yazici, Erhan Arslan, Kamil Gokce Tulaci, Haydar Ali Erken

**Affiliations:** ^1^Balikesir University, Faculty of Medicine, Department of Pathology, Balikesir, Turkey; ^2^Balikesir University, Faculty of Medicine Department of Otolaryngology, Balikesir, Turkey; ^3^Balikesir University, Faculty of Medicine Department of Physiology, Balikesir, Turkey

## Abstract

**Objective:**

To evaluate the effect of streptozotocin-induced experimental diabetes mellitus on p16, p53, Ki67, and Bcl2 expressions and histopathological changes in the tongue of the rats.

**Material and Methods:**

Twenty-two adult female Sprague-Dawley rats were used. The rats were randomly divided into 2 groups (*n* = 14) as control (C) (*n* = 8) and diabetic (DM) (*n* = 6). The rats in the DM group were given streptozotocin as a single intraperitoneal dose for induction of diabetes. Histopathological and immunohistochemical evaluations of formalin-fixed and paraffin-embedded tissue sections of the tongue were used.

**Results:**

Significant differences were observed between the DM group and the control group in terms of epithelial thickness, length of filiform papillae, and width of filiform papillae (*p* = 0.005, *p* = 0.001, and *p* = 0.006, respectively). There was no significant difference between the groups in terms of mononuclear inflammatory cell infiltration, capillary proliferation, and dysplasia (*p* = 0.204, *p* = 0.244, and *p* = 0.204, respectively). As a result of immunohistochemical studies, no significant difference was found between the groups in terms of p53, Ki67, and Bcl-2 expressions (*p* = 0.588, *p* = 0.662, and *p* = 0.686, respectively). A significant difference was found between the groups when p16 expression was evaluated (*p* = 0.006).

**Conclusions:**

In our study, streptozotocin-induced experimental diabetes mellitus induced p16 expression but did not show any difference in p53, Bcl-2, and Ki67 levels. It should be considered in the studies that the pathological changes at the early stages of the relationship between DM and oral cancer may be related to p16 expression; however, it may also be linked with p16-related aging process.

## 1. Introduction

Diabetes mellitus (DM) is a chronic metabolic disease with long-term complications affecting tissues such as the retina, kidney, heart, or peripheral nerve [1]. In addition, DM is associated with various oral conditions such as periodontal disease, tooth decay, geographic tongue, denture stomatitis, angular cheilitis, stomatitis, glossitis, fungal infections, and sensory changes [2]. The number of DM patients suffering from DM complications has been increasing day by day due to the prolongation of lifetime with technological advances and some other factors [3]. On the other hand, oral squamous cell carcinoma (OSCC) is the most common malignancy seen in the oral cavity of people throughout the world [4, 5]. As well as tobacco and alcohol, irregularity of oncogenes and tumor suppressor genes, epigenetic changes, and mitochondrial mutations play a role in the development of OSCC [5]. In recent epidemiological studies, DM has been shown to be a risk factor for both OSCC development and oral premalignant lesions [6, 7]. DM has also been reported to be a risk factor for oral premalignant lesions such as leukoplakia and lichen planus [7, 8]. On the other hand, there are studies reporting that there is no relationship between DM and oral premalignant lesions [6]. The tongue is the most common intraoral region for oral cancer, and tongue cancers are a public health problem that causes serious morbidity and mortality in many countries [9]. Although the incidence of tongue cancer appears to be stable or falling in some parts of the world, the incidence increases especially among men and women aged 18-44 years [9, 10].

Oral oncogenesis is a multistep process involving various histological changes such as hyperplasia, dysplasia, and carcinoma development [4]. As with other cancers, the development of squamous cell carcinoma is caused by the accumulation of mutations and epigenetic changes that alter the expression and function of oncogenes and tumor suppressor genes, leading to the acquisition of cancer properties such as cell death, increased proliferation, and resistance to induction [11]. There are two different pathogenic pathways in the development of squamous cell carcinomas of the oral cavity. The first is more common in people who use chronic alcohol and tobacco (either smoking or chewing). Mutations in this pathway are often in TP53 and genes that regulate the differentiation of squamous cells such as p63 and NOTCH1 [12]. The second tumor group includes oncogenic variants of human papilloma virus (HPV), in particular HPV-16. These tumors often show p16 overexpression. The prognosis of HPV-positive tumors is better than the prognosis of HPV-negative tumors [12].

Insulin-dependent DM (type I DM, IDDM) is thought to be an organ-specific autoimmune disease caused by the destruction of insulin-producing pancreatic *β*-cells [13]. Insulin deficiency results in reduced amounts of insulin receptor and consequently cell skeletal changes and reduced cell adhesion [4]. It has been asserted that decreased cell adhesion due to DM may cause an increased risk of oral cancer [14].

However, there is no clear evidence regarding the relationship between DM and oral/tongue cancer association. The aim of this study is to investigate the effect of streptozotocin-induced experimental DM on tumor suppressor genes p53 and p16, apoptosis marker Bcl-2, and cell proliferation marker Ki-67 and histopathological changes in the tongue of rats.

## 2. Materials and Methods

All experimental processes regarding the animals were in line with the National Institutes of Health Guidelines for the Care and Use of Laboratory Animals (NIH Publication No. 85-23). The approval of the University Local Ethical Committee was granted. Fourteen adult male Sprague-Dawley rats weighing 300-350 g were used. All the rats were kept in a 12 h light/dark cycle environment (lights on 7:00–19:00 h) at 22 ± 1°C and 50% humidity, and they were placed in translucent plastic cages (42 × 26 × 15 cm), each of which includes three or four rats. The rats had access to food and water *ad libitum*.

We divided the rats randomly into two groups (*n* = 14) as control (C) (*n* = 8) and diabetic (DM) (*n* = 6). Diabetes was induced through a single intraperitoneal (i.p.) injection of freshly prepared STZ (Sigma-Aldrich Co., Taufkirchen, Germany) solution (60 mg/kg body weight in 0.09 M citrate buffer, pH 4.8). The animals in the C group got the same volume of vehicle. Hyperglycemia was confirmed 48 h after STZ injection as a result of the measurement of tail vein blood glucose levels using a glucometer (Accu-Chek; Roche Diagnostics Co., Mannheim, Germany). Only animals with a plasma glucose level above 300 mg/dl were accepted as diabetic. The animals did not receive insulin treatment, and the animals were evaluated for the maintenance of the hyperglycemic state. At the end of the six-week experimental period, the rats were anaesthetized with ketamine/xylazine (90 and 10 mg/kg, respectively, i.p.).

In macroscopic examination, horizontally three parallel sections were evaluated for each tongue of rats. The tissues taken for histopathological evaluation as a result of necropsy after the induction of anesthesia were determined for 48 hours in 10% formalin solution. After passing through alcohol (70°, 80°, 90°, 96°, and 100°) and xylol series in routine tissue follow-up, they were buried in the paraffin blocks. 4 *μ*m sections were taken from each block, and preparations on the slide were made. Preparations for histopathological examination were stained with H&E and examined by a light microscope (Nikon Eclipse CI, Amsterdam, Netherlands). The tissues were evaluated by an experienced pathologist. The serial sections were examined twice in the light microscope. The tissues were histopathologically evaluated and scored in terms of mononuclear cell infiltration, dysplasia, and capillary proliferation as none (-), mild (+), moderate (++), and severe (+++) and in terms of dysplasia as none (-), mild/moderate (+), and severe (++) [15]. Tongue surface epithelium thickness, filiform papillae length, and filiform papillae width were measured (micrometer, *μ*m) using a software (Nis Elements 4.30) in a light microscope.

4 *μ*m tissue sections of formalin-fixed paraffin-embedded samples were applied immunohistochemical staining. p16 rabbit clonal antibody (prediluted 7 ml, Biotech, Kosice/Slovakia), Bcl-2 (alpha ab-1), p53 (DO7+BP53-12), and Ki67 (Richard Allan Scientific, Kalamazoo/USA) were used as primary antibodies. Staining was done on a Ventana Benchmark XT autostainer with the XT ultraView DAB Kit (Ventana Medical Systems, Roche Diagnostics Co., Mannheim, Germany). All slides were counter stained with haematoxylin. System controls were involved in order to exclude unspecific staining. In immunohistochemistry evaluation, the whole epithelial section was evaluated for staining, and nuclear and cytoplasmic staining was encountered the positive cells. The positive cells were evaluated and scored percentage of staining (0-100%).

### 2.1. Statistical Analysis

SPSS 20.0 program was used for statistical analysis. Data not indicating normal distribution were shown as a median (min-max) value. The median comparisons were made with the Mann-Whitney *U* test. *p* values < 0.05 were considered significant. The chi-square test was used to compare the ratios. For the correlation analysis, the Spearman correlation test was used for data not indicating normal distribution.

## 3. Results

**Figure 1:**
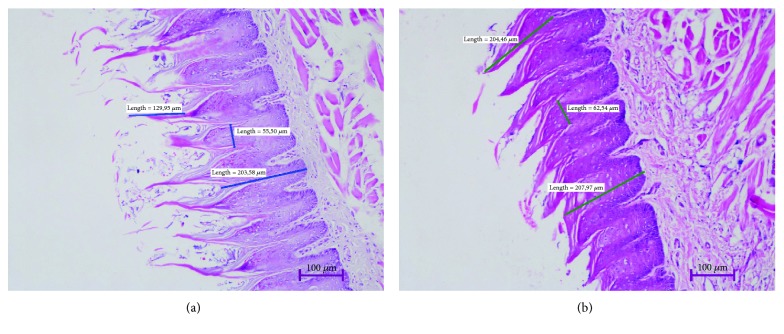
The difference between the structure of the filiform papillae and the epithelial thickness can be seen in the histopathological sections of the diabetic (a) and control (b) groups (H&E 100x).

The histopathological examination revealed that epithelial thickness decreased in the DM group, and the length and width of the filiform papillae decreased significantly compared to the control group (*p* = 0.005, *p* = 0.001, and *p* = 0.006, respectively) (Table 1, Figure 1). Significant dysplastic changes were not observed in both groups. No significant difference was observed between the groups in terms of mononuclear inflammatory cell infiltration, capillary proliferation, and dysplasia (*p* = 0.204, *p* = 0.244, and *p* = 0.204, respectively).

**Figure 2:**
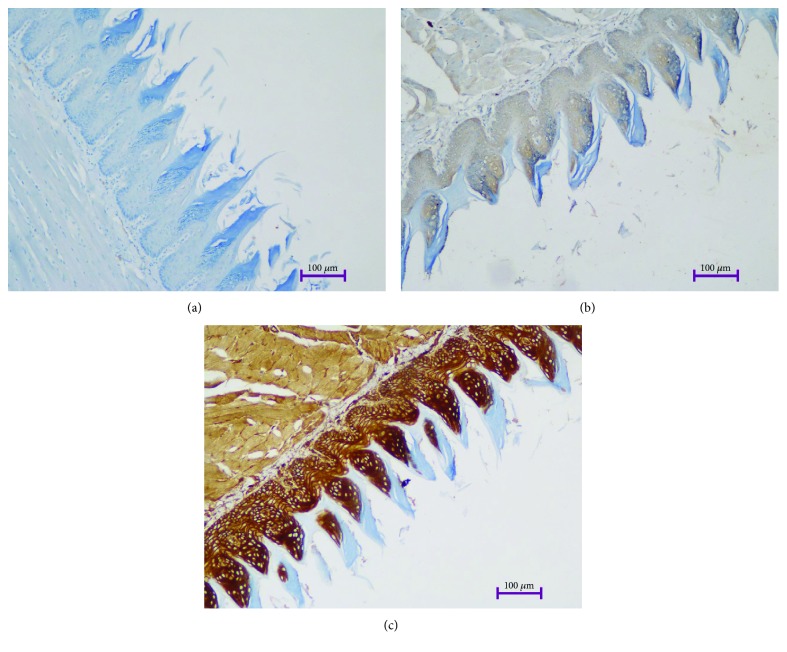
Negative (a), medium (b), and high (c) p16 expression in the tongue epithelium of diabetic rats (100x).

The p16 expression of the surface epithelium of the tongue sections of the DM group was significantly higher than the control group (*p* = 0.006) (Figure 2). No significant difference was found between the groups in terms of p53, Ki67, and Bcl-2 expressions (*p* = 0.588, *p* = 0.662, and *p* = 0.686, respectively) (Table 1).

## 4. Discussion

In this experimental study, we investigated the relationship between histopathologic changes occurring due to the expressions of tumor suppressor genes p53 and p16, apoptosis marker Bcl-2, and cell proliferation marker Ki-67 in the tongues of DM-induced rats. Significant histological changes in terms of epithelial thickness and filiform papillae structures were observed between DM-induced and control rats. It was found that DM significantly promoted the expression of p16 but did not make a difference in the expressions of p53, Bcl-2, and Ki67 which are important for oncogenesis. When the findings were examined in terms of the development of oral cancer and premalignant lesions in DM, it was found that the initial stage of the degeneration process is an increase in the p16 expression level, before chronic inflammation, epithelial thickness, capillary proliferation, and dysplasia.

Bcl-2 and bax proteins, associated with apoptosis, are two important effector genes during the apoptosis process. In some studies on human oral cancer biopsies, Bcl-2 levels have been reported to be increased compared to normal oral mucosa [16]. This corresponds to reduced apoptosis. In our study, there was no difference between the control group and the DM group in terms of Bcl-2 expression.

The expression of cell proliferation marker Ki-67 is higher in almost all stages of oral oncogenesis compared to normal ones [17]. In contrast, Ki67 expression in our study was low in the DM and normal group. These findings suggest that DM is performed during oral oncogenesis without initially affecting cell proliferation and Bcl-2-mediated apoptotic process.

p53 gene mutations were detected in a significant part of the OSCC. Inactivated mutant p53 protein is more stable than wild-type p53 protein, so its accumulation occurs early in oral neoplasia [5, 18]. The mutation accumulation gradually increases over successive oncogenic stages from hyperplasia to cancer [19]. Although there was no apparent epithelial dysplastic change in our study, there was a slight increase in the p53 expression of the DM group but no significant difference was found between the groups.

As one of the cell cycle regulators, p16 binds to CDK4 and inhibits cyclin D-CDK4 activity. Also, p16 is negatively regulated by pRB [20]. Although point mutations of the p16 gene are rare in head and neck cancers, alternative mechanisms for the elimination of the p16 function, including homozygous deletions or methylation of the 5-CpG promoter region of p16, are frequently identified [21]. Bova et al. showed in their study that cyclin D1 overexpression and loss of p16 expression were indicators of early relapse and low survival in squamous cell carcinoma of the anterior tongue [22].

The aging human body undergoes multiple physiological changes that lead to irreversible damage to organ systems. Aging is generally associated with a decrease in metabolic rate and glucose tolerance [23]. Hyperglycemia in aging may affect the onset of diabetes, and diabetes itself seems to accelerate the aging process [24, 25]. Recent studies highlight the effects of p16 on aging rather than tumor suppressor properties [26]. Liu et al. showed that p16INK4a tumor suppressor inhibited B cell neoplasms in a beneficial way while undesirably promoting T cell aging. In the same study, they suggested that p16 expression promoted aging while preventing cancer [27]. Similarly, in another study, cytotoxic cancer chemotherapy has been shown to increase the expression and cellular aging of p16 in T cells [28]. In our study, it was found that with increased p16 expression, decreased epithelial thickness, shorter filiform papillae width, and height in the DM group, DM was more likely to alter the morphological structure of the tongue and make it more prone to atrophy and aging.

Vairaktaris et al. suggested that p16 expression was gradually increased during oral oncogenesis in normal rats, but there was no significant difference in p16 expression between normal and diabetic animals during oral oncogenesis [29]. In our experimental study, cell cycle regulator p16 expression was found to be higher compared to normal rats before epithelial dysplastic changes occurred in DM.

The results of our study conducted to determine the relationship between DM and oral oncogenesis led us to think that early-onset degeneration occurring due to the absence of dysplasia, capillary proliferation, and MNL infiltration as well as not changing bcl2, p53, and Ki67 expressions might be related to p16 and its accelerating effect of aging (shortening of filiform papillae length and width and reduction in epithelial thickness).

The major limitation of our study is an experimental animal model which created diabetes irreversible. In addition, the duration of the experiment and observation was kept shorter than 6 weeks. In many other studies, epithelial dysplastic changes have been reported after 4 weeks, but it is clear that longer-term follow-ups will yield better results [4, 16].

In conclusion, in our study, significant histological changes were observed on the tongue between DM and normal rats. DM did not show any difference in p53, Bcl-2, and Ki67 levels while promoting p16 expression. The results of our study suggest that early pathological changes in the relationship between DM and oral cancer and p16-associated aging process may correlate with the p16 signaling pathway.

## Figures and Tables

**Table 1:** Histopathological and immunohistochemical findings of control and DM groups.

	Control	DM	*p*
Epithelial hyperplasia (*μ*m)^∗^	235.0 (136.2-403.5)	120.4 (60.3-277.6)	**0.005**
Length of filiform papillae (*μ*m)^∗^	221.8 (162.0-642.9)	99.5 (67.2-211.8)	**0.001**
Width of filiform papillae (*μ*m)^∗^	87.2 (61.5-159.6)	55.6 (21.0-80.5)	**0.006**
Capillary proliferation (0/1/2/3)^#^	1/7/0/0	1/9/4/0	0.244
Dysplasia (0/1/2/3)^#^	6/2/0/0	6/8/0/0	0.204
MNL infiltration (0/1/2/3)^#^	5/3/0/0	8/6/0/0	0.204
p16 (%)^∗^	40 (10-70)	90 (15-90)	**0.006**
p53 (%0/%1/%2)^#^	3/4/1	3/7/4	0.588
Ki67 (%0/%1)^#^	4/4	5/9	0.662
Bcl-2 (%0/%1/%2)^#^	5/3/0	7/6/1	0.686

^∗^As the parameters did not show a normal distribution, the data were shown as median (min-max) values. Mann-Whitney *U* test was used for comparisons; ^#^Chi-square test was used to compare ratios.

## Data Availability

The data used to support the findings of this study are available from the corresponding author upon request.
